# Fabrication of Zirconia-Reinforced Lithium Silicate Ceramic Restorations Using a Complete Digital Workflow

**DOI:** 10.1155/2015/162178

**Published:** 2015-10-05

**Authors:** Sven Rinke, Matthias Rödiger, Dirk Ziebolz, Anne-Kathrin Schmidt

**Affiliations:** ^1^Dental Practice, 63456 Hanau, Germany; ^2^Department of Prosthetics, University Medical Center Göttingen, 37075 Göttingen, Germany; ^3^Department of Restorative Dentistry and Periodontology, University Medical Center Leipzig, 04103 Leipzig, Germany

## Abstract

This case report describes the fabrication of monolithic all-ceramic restorations using zirconia-reinforced lithium silicate (ZLS) ceramics. The use of powder-free intraoral scanner, generative fabrication technology of the working model, and CAD/CAM of the restorations in the dental laboratory allows a completely digitized workflow. The newly introduced ZLS ceramics offer a unique combination of fracture strength (>420 MPa), excellent optical properties, and optimum polishing characteristics, thus making them an interesting material option for monolithic restorations in the digital workflow.

## 1. Introduction

As a result of continuing development in computer-based dental technologies, new opportunities for clinical workflows and the manufacturing of dental restorations have been introduced to the dental field [1]. Over the last decade, computer-aided design/computer assisted manufacturing (CAD/CAM) of dental restorations has become an established fabrication process, especially for all-ceramic restorations [2]. With the more recent introduction of intraoral scanning systems, digital techniques are already capable of replacing conventional workflows [3, 4]. Meanwhile, a number of clinical trials have demonstrated that single-tooth restorations fabricated in a completely digitized workflow have a fitting quality that is equal to or better than conventionally fabricated restorations [4–9]. Moreover, compared with conventional impressions, digital impressions can be more time-efficient and improve the treatment comfort [10, 11].

Apart from the constant improvement of digital technologies, new restorative materials that are optimized for CAD/CAM processes lead to a further development of digital workflows [2, 3]. Research focuses on the development of materials that offer a combination of adequate translucency, improved mechanical strength, and optimized timesaving machining [12].

Among others, a new group of machinable ceramics has recently been introduced for CAD/CAM techniques: zirconia-reinforced lithium silicate (ZLS) ceramics (Celtra Duo, Dentsply DeTrey, Konstanz, Germany; Suprinity, Vita Zahnfabrik, Bad Säckingen, Germany). According to the manufacturers, these materials offer mechanical properties ranging from 370 to 420 MPa. Thus, they are comparable with the clinically well-proven lithium disilicate (Ls2) glass ceramics [13]. The values for mechanical properties are approximately three times higher than those determined for traditional leucite-reinforced glass ceramics (IPS Empress, Ivoclar Vivadent, Schaan, Liechtenstein). According to the manufacturers, in vitro testing revealed a Weibull modulus of 8.9 for this material group. The high Weibull modulus indicates a uniform material quality. In addition to the high load capacity values, this material quality is an indicator for the reliability of a material. The improved strength and reliability are reached by the addition of 8–10 wt% of zirconium oxide [14]. After crystallization, the presence of zirconia causes a homogeneous texture to form with a mean grit size of approximately 0.5 to 0.7 *µ*m. The formed crystals are 4 to 8 times smaller than lithium disilicate crystallites. Therefore, ZLS ceramics consist of a dual microstructure:

- very fine lithium metasilicate and lithium disilicate crystals (average size: 0.5–0.7 *µ*m); this is the main difference from Ls2 ceramics, which only contain lithium disilicate crystals;
- glassy matrix containing zirconium oxide in solution.

This dual microstructure is achieved in a two-step process. The material is delivered in a precrystallized stage, containing only lithium metasilicate crystals. In its precrystallized phase, the material is easy to machine. After the water-cooled milling process and the finishing of the restoration, the final dual lithium silicate microstructure is reached during an 8-minute firing process at 840°C [12].

The result is a very fine microstructure that allows a high flexural strength while simultaneously providing a high percentage of glassy matrices. These structural effects provide the ceramics with good optical and polishing properties. Today, these materials are available as industrially prefabricated blanks for various CAD/CAM systems (e.g., Cerec, Sirona, Bensheim, Germany; Artica, KaVo, Leutkirchen, Germany; and Ceramill, Amann Girrbach, Pforzheim, Germany) in various shades and translucencies.

This report describes a treatment with monolithic ceramic restorations made from a ZLS ceramic (Suprinity, Vita Zahnfabrik, Bad Säckingen, Germany) using a completely digital workflow.

## 2. Case Presentation

**Figure 1:**
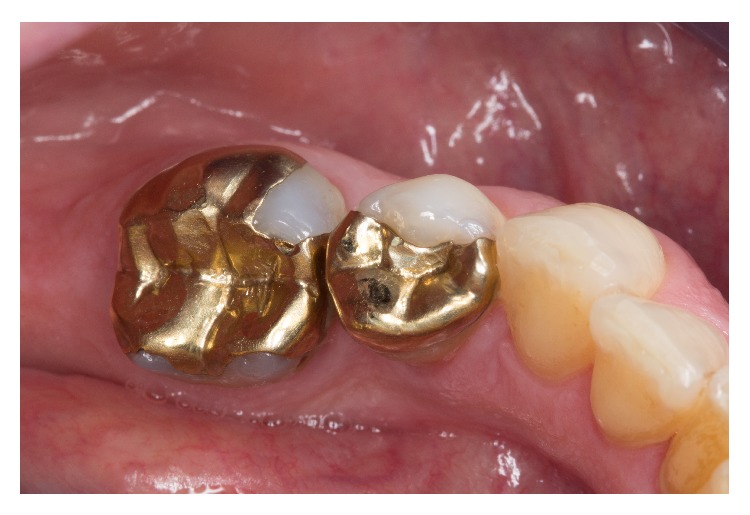
Initial situation with insufficient cast partial crowns on teeth 45 and 46.

A 42-year-old woman required prosthetic treatment of the lower right second premolar and the first molar. For these restorations, repeated clinical intervention had been necessary due to the loss of retention and secondary caries (Figure 1).

Both abutment teeth were vital, and the overall periodontal situation was stable. The patient opted for a replacement of the cast gold restoration by a monolithic all-ceramic crown on the first molar and an all-ceramic partial crown fabricated from a ZLS ceramic on the premolar.

**Figure 2:**
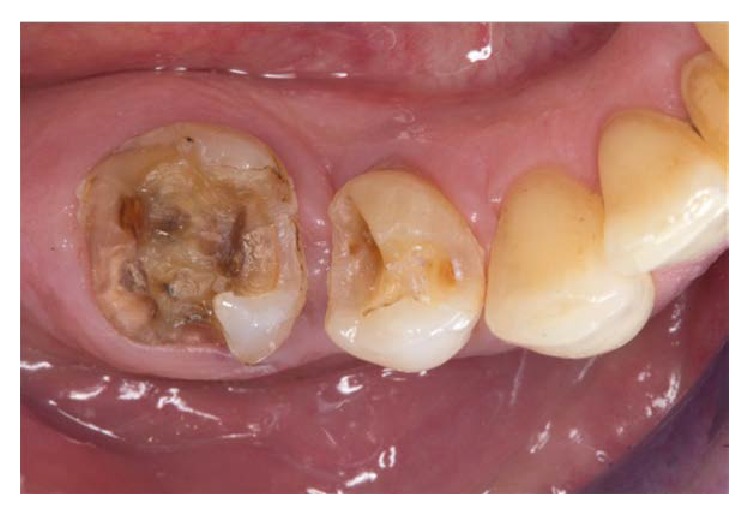
Clinical situation after removal of restoration and caries.

The next clinical appointment started with the shade selection using a conventional shade guide (Vitapan classic, Vita Zahnfabrik, Bad Säckingen, Germany). After the application of local anesthetics (UltraCain DS, Sanofi Aventis, Frankfurt, Germany), the existing posterior crowns were detached, and the caries was completely removed (Figure 2). The core build-ups were positioned adhesively (Core-Up OptiMix, Kaniedenta, Herford, Germany).

Due to the large defect, tooth 46 was to be restored with an all-ceramic crown and tooth 45 was to receive a ceramic partial crown. Preparation for both restorations was performed according to the recommendations of Ahlers et al. (2009) [15], avoiding sharp edges and preparing round surfaces wherever possible. A minimum wall thickness of 1.2 to 1.0 mm in the occlusal area was maintained. The preparation limit of the full coverage crown was carried out as a shoulder preparation with an internal rounded line angle and a cutting depth of 1.0 mm.

**Figure 3:**
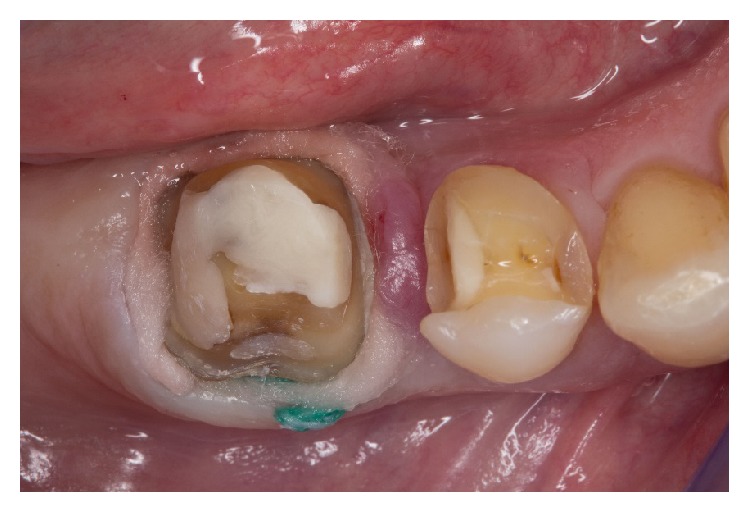
Preparation of teeth for digital impression-taking. After application of the retraction cords, a top layer of cotton coil impregnated with epinephrine for hemostasis is applied.

Before the digital impressions were taken, two layers of nonimpregnated retraction cords (sizes 0 and 2) (Ultradent Products, Köln, Germany) were placed on the first molar. By setting a second cord with a larger diameter directly over the first, a V-shaped pack was created, which provided a physical lateral displacement of the tissues. Finally, a top layer of cotton coil impregnated with epinephrine for hemostasis (Gingi-Pak, Belport Company, Camarillo, USA) was looped around the tooth (Figure 3). After waiting for approximately 10 minutes until the sulcus was expanded adequately, only the hemostatic cotton-coil layer was removed. The teeth were air-dried, while the retraction cords remained in the sulcus. Due to a supragingival preparation of tooth 45, no preparing steps were required here for digital impression-taking. Intraoral scanning was performed with a powder-free technology (Trios, 3 Shape, Copenhagen, Denmark), which uses ultrafast optical sectioning and confocal microscopy. This system recognizes variations in the focus plane of the pattern over a range of focus plane positions while maintaining a fixed spatial relation between the scanner and the scanned object. Furthermore, a scanning speed of up to 3,000 images per second reduces the influence of relative movement between the scanner probe and teeth. By analyzing a large number of pictures, the system can instantly create a final digital 3D model to reflect the real configuration of teeth and soft tissue.

**Figure 4:**
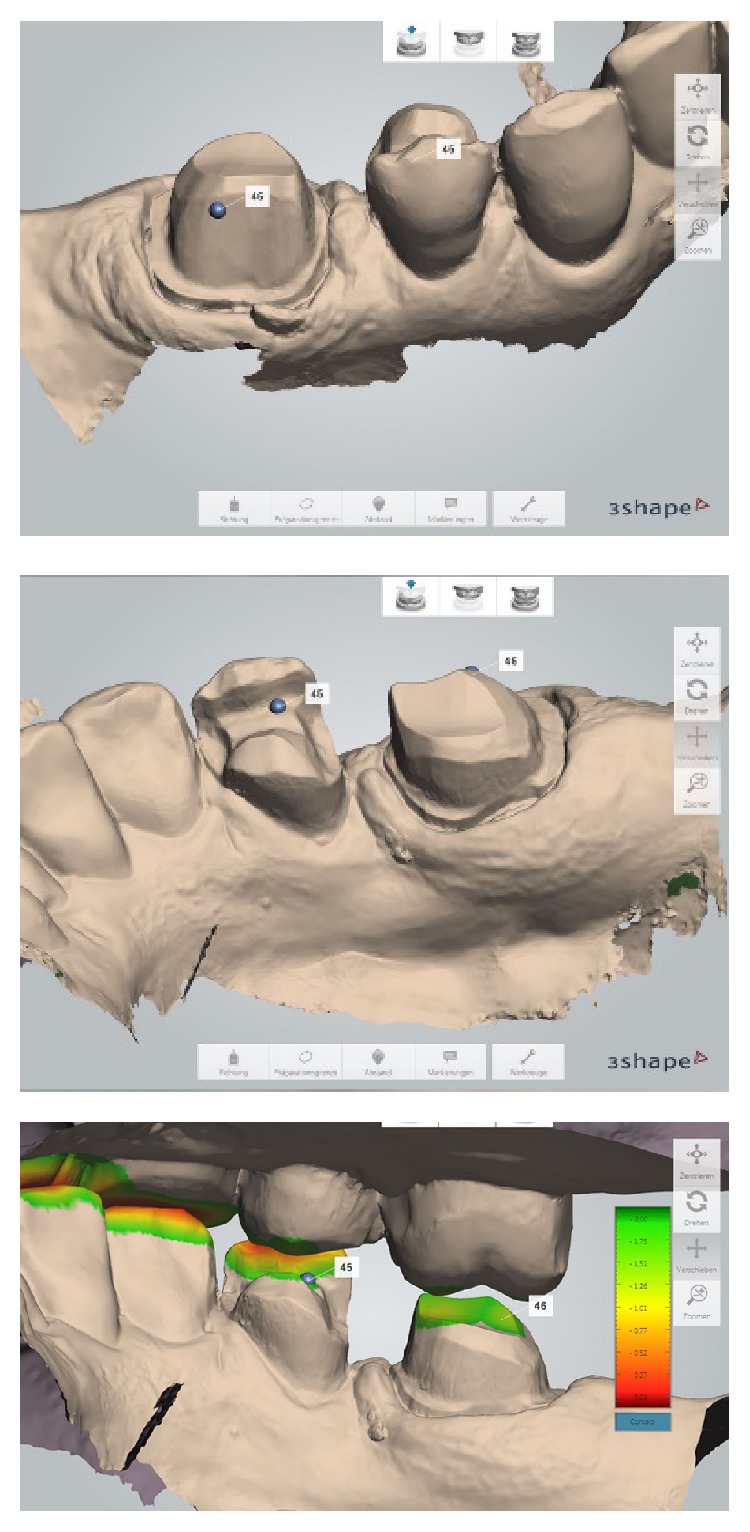
Detailed views of the intraoral scanning process and the measuring function for the substance reduction after digital bite registration.

First, the lower right quadrant of the mandible was scanned, followed by data acquisition in the upper right quadrant. A buccal scan was taken when the patient closed into an intercuspal position (Figure 4). The system implemented the digital registration to create a relation for 3D occlusion. The operation of the TRIOS scanner is relatively simple. The scanner can be held at various distances from the tooth. Either closely over the tooth or 2 to 3 cm away, the distances will affect neither the focus nor the capturing of images. The 3D profiles of the teeth and gingiva are generated simultaneously, while the scanner tip gradually moves above them.

Afterwards, the data and color information were forwarded to the dental lab. The TRIOS system is an open system that can export 3D data as an STL file or a proprietary file. The STL file can be used with various CAD/CAM systems. The proprietary encrypted file can only be designed by 3 Shape's specific CAD software and the 3 Shape Dental System. At the end of this appointment, the preparations were restored with directly produced temporaries (Luxatemp, DMG, Lübeck, Germany), which were luted with a eugenol-free luting agent (Temp Bond Clear, Kerr Hawe, Karlsruhe, Germany).

**Figure 5:**
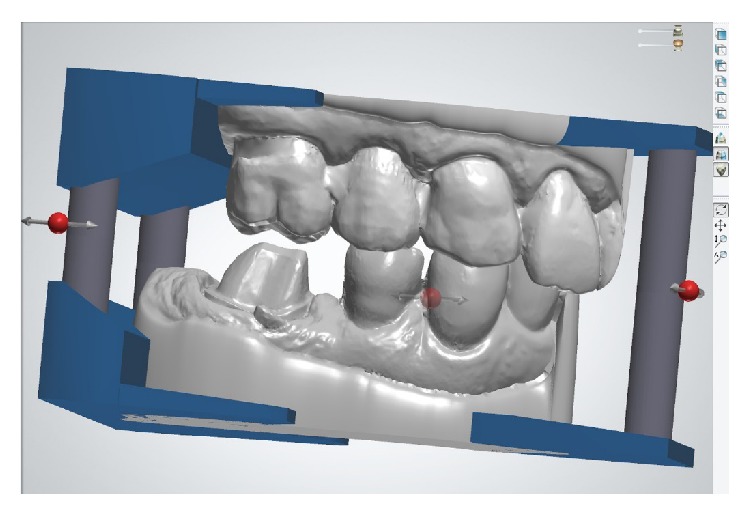
Virtual design of the working models (Model Builder 2013, 3 Shape, Copenhagen, Denmark).

At the dental laboratory, the digital set of data from the scanning procedure was used to create virtual working models with retrievable dyes using computer software (2013 Model Builder, 3 Shape, Copenhagen, Denmark) (Figure 5).

**Figure 6:**
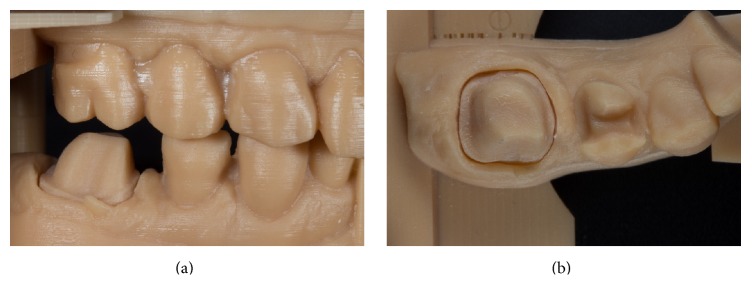
Generatively manufactured working model.

After checking the occlusal contacts and the preparation margins, the digital file of the working model was transferred to a production center (Innovation Meditech, Unna, Germany). Dental models made of a beige high-performance resin (80–84 Shore D) were produced in a generative manufacturing process (LED-scanning technology based on STL files) and returned to the dental laboratory within 48 hours (Figures 6(a) and 6(b)).

**Figure 7:**
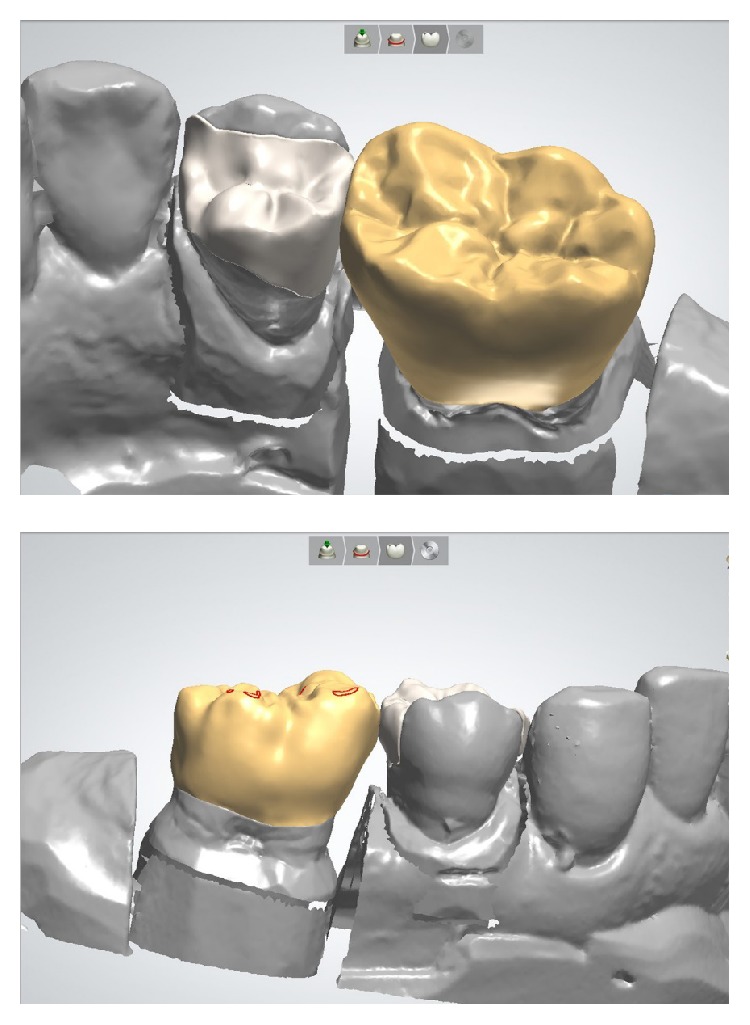
Construction of monolithic crown and partial crown restorations with CAD software (DentalDesigner 2013, 3 Shape, Copenhagen, Denmark).

After exploring the STL files for the production of the working models, the design process for the fully anatomic restorations was initiated with a CAD software package (DentalDesigner 2013, 3 Shape, Copenhagen, Denmark) (Figure 7). The minimum material thickness was set to 1 mm; the cement spacer thickness was set to 30 *µ*m. Finally, the designed restorations were exported as STL files.

The restorations were then milled as a full-contour monolithic ZLS crown/partial crown using a 5-axis compact milling unit (Ceramill motion 2, Amann Girrbach, Pforzheim, Germany) in a wet grinding process.

**Figure 8:**
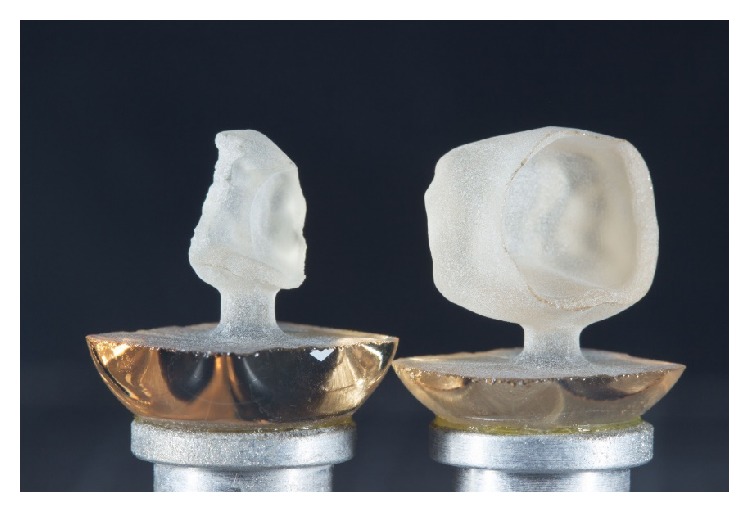
ZLS crown and partial crown directly after the milling process.

For the milling of the partial crown, the material type HT (high translucency) was selected to generate a pronounced chameleon effect, while the full-coverage crown was milled from type T (translucent) (Figure 8).

**Figure 9:**
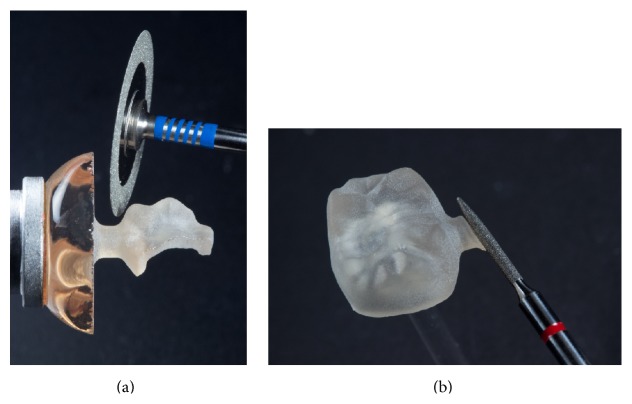
Removal of the fixation pin and shaping of the ground ceramic restorations with water-cooled diamond instruments.

After the machining of the restorations, the fixation bar of the restoration was ground with water-cooled diamond instruments. Afterward, the occlusal surfaces were also reworked with water-cooled diamond instruments (Figures 9(a) and 9(b)).

The next step was the internal adjustment and the adjustment of the proximal contacts on the digitally produced working model, followed by the occlusal adjustment of the restorations. As the precrystallized stage of the material is easy to process, at this point, the contour and the occlusal surface should be worked out in as much detail as possible. However, the material strength required for clinical application (>420 MPa) is reached only after crystallization firing. Here, two procedures are possible: (1) combination firing (i.e., crystallization in combination with stains firing) at 840°C for 8 minutes followed by a slow-cooling or (2) separate crystallization firing (840°C) as the first step and stains firing (800°C) in a second step. Irrespective of the selected procedure, the restorations have to be cleaned prior to crystallization in an ultrasonic bath for 10 minutes or by using a steam jet unit. ZLS ceramics must not be air-abraded with Al_2_O_3_ or abrasive beads.

**Figure 10:**
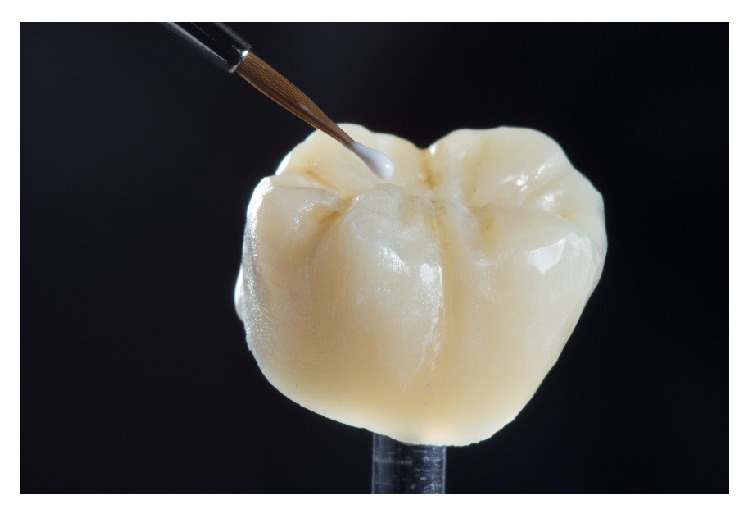
Individual coloring of the crystallized ZLS restoration with special staining liquids.

**Figure 11:**
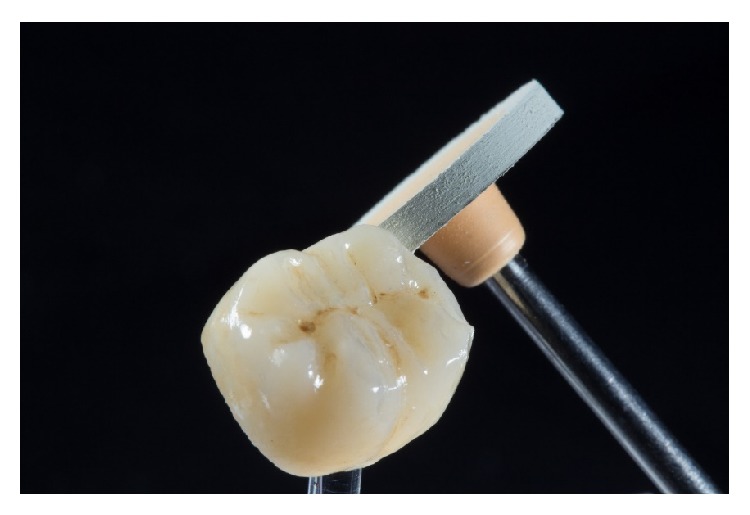
Final polishing.

**Figure 12:**
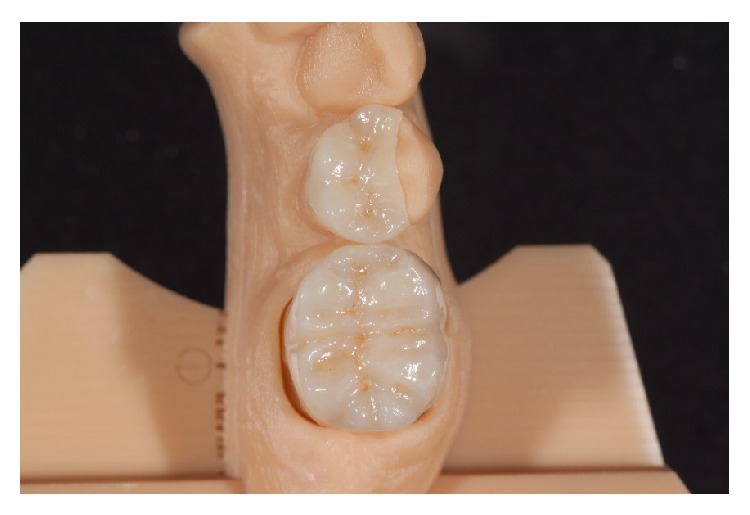
Final fit checking with proximal and occlusal contacts on the digitally fabricated working model.

In the present case, the two-step procedure was chosen. After the first crystallization firing, the restorations were further individualized by using two layers of stains in two separate firing cycles at 800°C (Figure 10). The restorations were then mirror-finished with diamond-impregnated silicone instruments and polishing pastes (Figure 11). Finally, occlusion and proximal contacts were checked and adjusted on the digitally fabricated working models (Figure 12).

**Figure 13:**
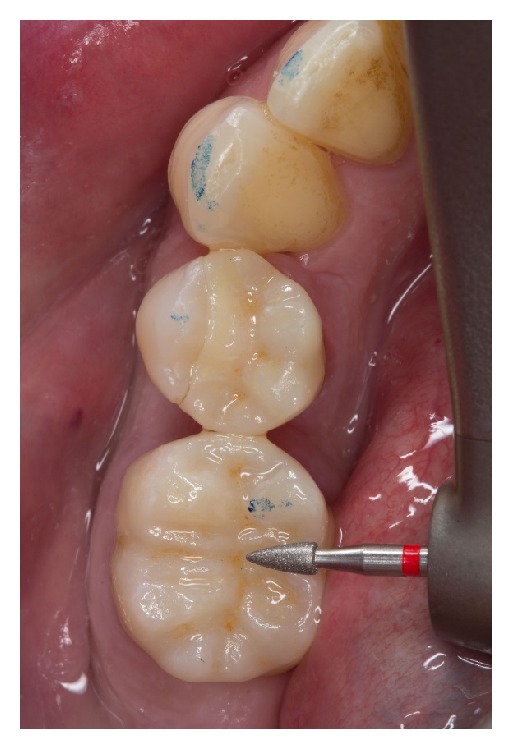
Occlusal adjustments of the restoration during try-in with a fine-grit-size diamond instrument (8390.314.016, Gebr. Brasseler, Lemgo, Germany).

Two weeks after taking the impressions, the restorations were tried in with a try-in gel (Calibra Try-In Paste, Dentsply DeTrey, Konstanz, Germany) to verify the fitting accuracy and the correct match of the shade. First, each restoration was tried in separately to check the correct marginal fit. If the fit was considered good, the restorations were tried in together, and proximal contacts were checked. Due to the high final strength (420 MPa) of the ceramic restorations after glazing, a careful checking of the occlusal contacts was possible. Selective adjustment could be performed with water-cooled fine diamond instruments (8390-016, Gebr. Brasseler, Lemgo, Germany) (Figure 13).

**Figure 14:**
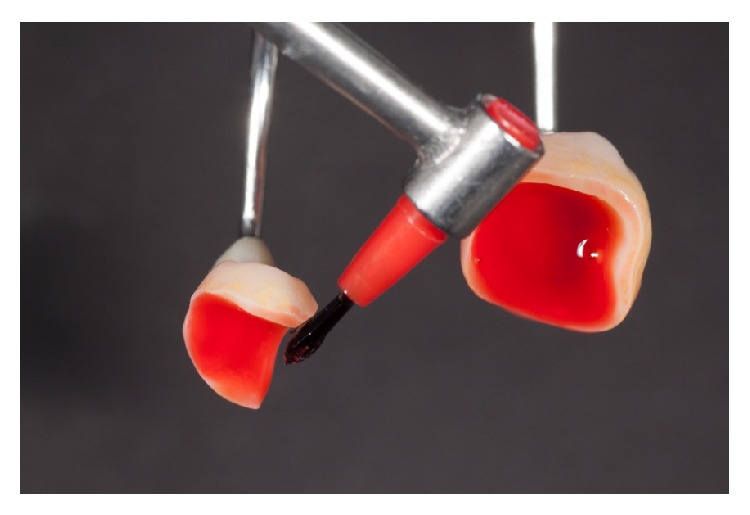
Conditioning of the cementation surface with 5% hydrofluoric acid for 20 seconds.

After removal of the restorations, the adjusted areas should be polished carefully with diamond-impregnated silicone instruments (Vita Suprinity Polishing Set Clinical, Vita Zahnfabrik, Bad Säckingen, Germany). Prior to adhesive luting, the restoration surfaces intended for bonding were conditioned with 5% hydrofluoric acid (Vita Ceramics Etch, Vita Zahnfabrik, Bad Säckingen, Germany) for 20 seconds (Figure 14).

**Figure 15:**
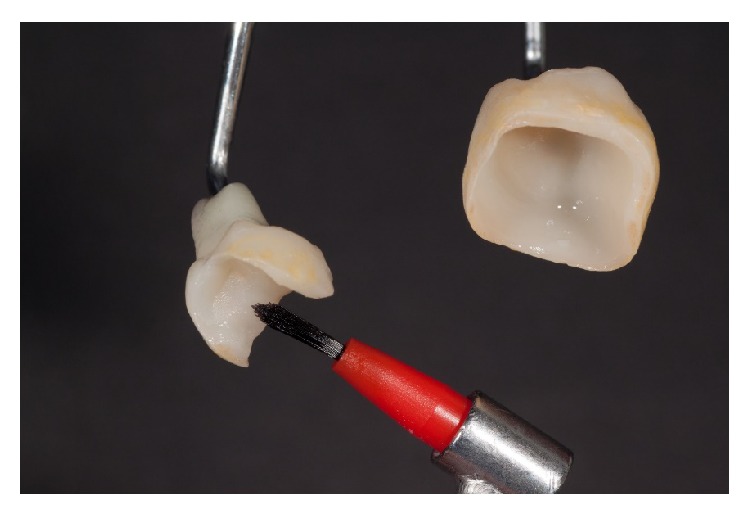
Application of silane on the cementing surfaces after conditioning with 5% hydrofluoric acid.

Afterward, the restorations were thoroughly rinsed with water until all acid residue was removed. For better control of cleansing, a stained etching gel is preferred. After drying the etched restorations, a silane material (Calibra Silane, Dentsply DeTrey, Konstanz, Germany) was applied (residence time: 1 minute) (Figure 15).

After cleaning the teeth with pumice and chlorhexidine solution, the teeth were isolated using a rubber dam. The enamel was conditioned with 37% phosphoric acid for 30 seconds, while the exposed dentine was etched for 15 seconds. Adjacent teeth should be protected with cellophane matrices prior to application of the etching gel. If the proximal surfaces of the adjacent teeth are conditioned unintentionally, cement excess on these surfaces will cause difficulties in the remainder of the process.

A dual-curing one-step adhesive (XP Bond and Self-Curing Activator, Dentsply DeTrey, Konstanz, Germany) was applied on both teeth and restoration surfaces, and then the adhesive was air-thinned. A thin layer of a dual-curing transparent resin cement (Calibra automix transparent, Dentsply DeTrey, Konstanz, Germany) was immediately applied to the restorations that were subsequently seated. After precuring of the resin cement for 3–5 seconds on the lingual and the buccal sides, the excess cement was removed with an explorer and dental floss in the proximal areas. The final light curing of the luting agent was performed for 40 seconds on each side of the restoration (buccal/occlusal/lingual). Finally, the occlusal contacts were checked and adjusted where necessary. Margins were finished and polished with fine-grit-size diamond instruments and diamond-impregnated polishers. Due to the microstructure of the ZLS ceramics with nanoscaled crystals, intraoral polishing could be performed very time-effectively with the recommended 2-stage polishing system (Vita Suprinity Polishing Set Clinical, Vita Zahnfabrik, Bad Säckingen, Germany). Diamond pastes for intraoral polishing (e.g., Direct Dia Paste, Shofu Dental, Ratingen, Germany; Optra Fine HP Polishing Paste, Ivoclar Vivadent, Schaan, Liechtenstein) are especially suitable for final polishing.

**Figure 16:**
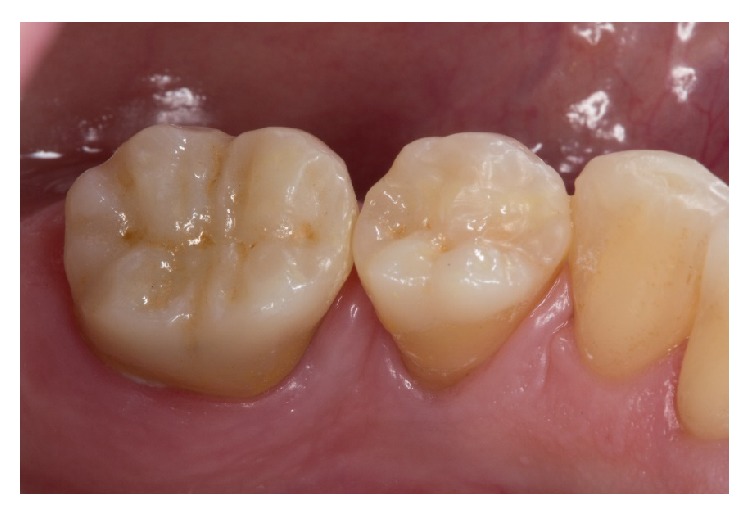
Situation 2 weeks after adhesive luting of the restorations. The good light-optical properties of ZLS allow a perfect color match.

The patient was reexamined two weeks after cementation and reported no postoperative sensitivity. The postoperative tissues were healthy, and both restorations showed a good shade adaption. The patient was very satisfied with the treatment (Figure 16).

## 3. Discussion

Since the 1980s, computer-aided design and computer-aided manufacturing (CAD/CAM) have been employed in the fabrication of restorations, especially for ceramic crowns and fixed dental prostheses (FDPs).

The development of new intraoral scanning devices and new high-strength and reliable ceramic materials has drawn comprehensive attention from dentists and dental technicians. A number of publications have indicated that digital techniques are capable of replacing conventional workflows for at least single-tooth restorations and short-span FPDs [1, 3–9]. This claim is supported by the clinical experience documented in the present case report.

Three major developments have been crucial. 

*Intraoral Scanner*. Earlier scanning systems required the time-consuming application of a scanning powder. With the introduction of powder-free scanning systems such as the one applied in the present case report, the scanning process is simplified and shortened [1, 3, 4, 9]. Moreover, the open gateways of modern scanning systems allow for easy data transfer, thus granting access to various production procedures. Therefore, a large variety of materials can be processed digitally [3, 4].

*Digitally Produced Models*. Working models are essential for the fabrication of restorations, especially for veneered or more complex restorations. Thus, the production of precise working models is a precondition for a 100% digitized workflow for prosthetic restorations exceeding single-tooth restorations [1, 3, 9]. The further development of manufacturing technologies for digital working models is important for progress and improvement. 

*Innovative Materials*. Further developments in ceramic materials aim to create an ideal combination of strength and optical properties [2]. Due to their combination of strength and translucency, ZLS ceramics offer ideal preconditions for the fabrication of monolithic restorations that are characterized by staining only [12]. The omission of ceramic veneering eliminates the risk of veneering ceramic fractures and moreover allows time-saving and efficient fabrication of the restorations [2, 12]. Due to its special microstructure, this material group is easy to polish. Furthermore, considering the good optical properties with their so-called chameleon effect, these materials allow for chairside production of ceramic restorations [12]. Nevertheless, it is a limitation of these new materials that data from clinical studies are still missing.

However, despite considerable progress in the further development of digital workflows, the financial investments for both the practice (intraoral scanner) and the dental laboratory (CAD software, milling unit) are high in comparison with conventional workflows [3].

## 4. Conclusions

ZLS ceramics offer a good combination of high strength and outstanding optical properties. Thus, these materials are interesting for the fabrication of monolithic restorations. A mere CAD/CAM fabrication of these restorations is possible with the components available (intraoral scanner, CAD software, milling unit, and technology for model fabrication). However, although ZLS ceramics show a positive combination of properties that were verified in laboratory studies, the indication should be chosen with strict observation of the material-specific processing instructions. This attention to processing instructions is especially important regarding the necessary minimum wall thickness and required adhesive luting. Results from clinical studies are needed to verify the positive results from these initial clinical experiences.
